# Trends in the Management of Keratoconus in Australia

**DOI:** 10.1007/s44402-026-00096-2

**Published:** 2026-05-19

**Authors:** Richard Kha, Himal Kandel, Stephanie Watson

**Affiliations:** Save Sight Institute, Faculty of Medicine and Health, The University of Sydney, Sydney, New South Wales Australia

**Keywords:** Australia, Contact lenses, Corneal transplant, Crosslinking, Keratoconus, Keratoplasty

## Abstract

**Purpose:**

Over the past few decades, management options for keratoconus have evolved to include corneal crosslinking (CXL), keratoplasty and advanced contact lenses. This study aimed to describe trends in the use of various keratoconus management techniques in Australia.

**Methods:**

This cross-sectional study extracted data on the use of keratoconus management techniques in Australia from various data sources. Corneal graft and national CXL data were obtained from the Australian Corneal Graft Registry, Medicare Benefits Schedule and Save Sight Keratoconus Registry. Data on the number and proportion of rigid gas permeable (RGP) and scleral contact lenses were collected from the International Contact Lens Prescribing Survey and contact lens manufacturers. The number of RGP and scleral contact lenses, keratoplasty and CXL used for managing keratoconus in Australia were analysed.

**Results:**

Corneal grafts for keratoconus decreased from 3170 in 2001–2010 to 2797 in 2011–2020. This coincided with increased use of CXL, as well as RGP and scleral contact lens use. Since 2007, the proportion of RGP lenses sold in Australia has increased from 5% to 18% of all lenses. Over the past three decades, the estimated number of RGP and scleral lenses sold for the treatment of keratoconus has doubled. CXL use has increased and was more common in males and patients aged 15–24 years, with variability in corneal CXL rates across each state.

**Conclusions:**

In Australia, there is a decreasing trend in keratoplasty for keratoconus along with increases in CXL and contact lens use. Future studies should evaluate outcomes associated with the change in keratoconus management patterns.

Key Points

- Fewer corneal transplants coinciding with increased corneal crosslinking and contact lenses suggest a shift towards earlier, less invasive treatment to stabilise and manage keratoconus, avoiding the need for graft surgery.
- Younger Australian adults, particularly males, have higher rates of corneal crosslinking, and regional differences in crosslinking rates suggest variation in referral patterns, service availability and approaches to early keratoconus care.
- Future studies should assess both objective and patient-reported outcomes associated with the change in keratoconus management patterns, including real-world outcomes of emerging treatments such as corneal allogeneic intrastromal rings and specialised contact lenses.

## Introduction

Keratoconus is a common corneal ectatic disease characterised by bilateral, progressive corneal thinning and protrusion that results in irregular astigmatism [1]. It typically occurs in young adults, starting from the second decade of life and remains a significant cause of vision impairment [2]. The prevalence of keratoconus is highly variable, in part due to the heterogeneity of the definition of keratoconus used in epidemiological studies. Keratoconus was originally described as a rare disease, with a reported prevalence of approximately 1 per 2000 individuals [3]. Recent data has found that keratoconus may be more prevalent than previously thought. A meta-analysis of 15 studies published in 2020 reported that the global prevalence of keratoconus was 1.4 per 1000 [4]. Newer diagnostic technologies such as Scheimpflug tomography, epithelial mapping and artificial intelligence (AI) tools have likely contributed to the increase in keratoconus prevalence by allowing the disease to be detected at an earlier stage. This shift is evident in population-based studies that define keratoconus using Scheimpflug imaging, such as the Raine study in Australia, which reported a prevalence of 12 per 1000, and an investigation in Riyadh, which found a prevalence of 1 per 21 patients, both higher than historical estimates [5, 6]. Diagnostic efforts now focus on multimodal models that combine Scheimpflug tomography with epithelial mapping and AI-based deep learning systems [7]. Such systems accurately detect manifest keratoconus and also display potential for uncovering subclinical keratoconus [8, 9]. As the prevalence of keratoconus rises, optimising timely, effective treatment becomes increasingly critical. Over the past few decades, treatment options for keratoconus have evolved. According to the Global Consensus on Keratoconus and Ectatic Diseases, the main goals of keratoconus treatment include halting disease progression and visual rehabilitation [1]. Current management techniques involve spectacles, rigid gas permeable contact lenses (RGP), keratoplasty and corneal crosslinking (CXL) [10]. Keratoplasty is one of the most common procedures worldwide for the treatment of keratoconus in many countries [11, 12]. In 2003, Wollensak et al. described CXL using the Dresden protocol to halt disease progression and reduce the need for keratoplasty [13]. The implementation of CXL and improved contact lens fitting has led to a progressive decrease in the use of keratoplasty for keratoconus in some countries, particularly in the developed world [11, 12, 14]. CXL has been available in Australia for a decade and was added to the Medicare Benefits Schedule on 1 May 2018. Prior to this addition, patients with keratoconus were required to pay significant out-of-pocket costs for the procedure. The cost and lack of coverage of CXL by government and/or health insurance are barriers to the widespread use of CXL in many countries.

There are no comprehensive Australian data on keratoconus management and burden, including the use of contact lenses, keratoplasty and CXL. Due to the reported high prevalence of keratoconus in Australia, its impact on the quality of life (QoL) [15, 16] and health care costs, it is important to understand trends in keratoconus and its management to enable improvements in outcomes. Hence, this study aimed to evaluate trends in the use of contact lenses, keratoplasty and CXL for managing keratoconus in Australia from 2000 to 2024.

## Materials and Methods

This cross-sectional study collected information on trends in keratoconus and its management in Australia from various public and private data sources. Corneal graft data were obtained from the Australian Corneal Graft Registry (ACGR), which included statistical analysis of corneal transplantation practices in Australia [17]. The ACGR aims to collect data on all corneal grafts performed in Australia, with participating surgeons reporting their patients' corneal graft outcome data. Only corneal grafts where the primary surgical indication was recorded as keratoconus were included in the study. The Medicare Benefits Schedule (MBS) was used to obtain the number of CXL procedures performed in Australia since the addition of the procedure to the MBS in May 2018. The MBS is a list of healthcare services subsidised by the Australian Government, and contains national information about all the subsidised CXL procedures; patients treated in private and public settings can access subsidised treatment [18]. Privately funded non-Medicare eligible patients or non-subsidised cross-linking data are not captured by the MBS. Additional data on the number of CXL procedures performed in Australia were obtained from the Save Sight Keratoconus Registry (SSKR), an online multinational database for tracking keratoconus outcomes in patients from various public and private sites [19]. Data from Australian sites from the SSKR were included in this study, whereas missing or non-Australian data were excluded. RGP contact lenses are used to correct irregular astigmatism in keratoconus, while scleral contact lenses are typically used when corneal ectasia with irregular astigmatism cannot be corrected by RGP lenses. The number and proportion of RGP and scleral contact lenses sold per year in Australia were determined using data from the International Contact Lens Prescribing Survey from 2001 to 2024 [20]. The International Contact Lens Prescribing Survey has collected annual data on contact lens prescription in various countries since 2001 [20]. Data regarding Australian prescriptions for RGP and scleral lenses were included in this study. Additional data on the number of contact lenses sold for keratoconus were attained by asking contact lens manufacturers and from the MBS data. The findings on RGP contact lens use, keratoplasty and CXL in Australia were analysed with descriptive statistics. For data from the Save Sight Keratoconus Registry, ethics approvals were obtained from the Sydney Local Health District Ethics Review Committee (RPAH Zone; Protocol No X20-0487 and 2020/ETH02676), and from the public and the ethics committee of the Royal Australian and New Zealand College of Ophthalmologists (HREC Ref. 50.14) for the private sites. The study was performed in accordance with the principles outlined in the Declaration of Helsinki.

## Results

### Keratoplasty for Keratoconus

**Fig. 1:**
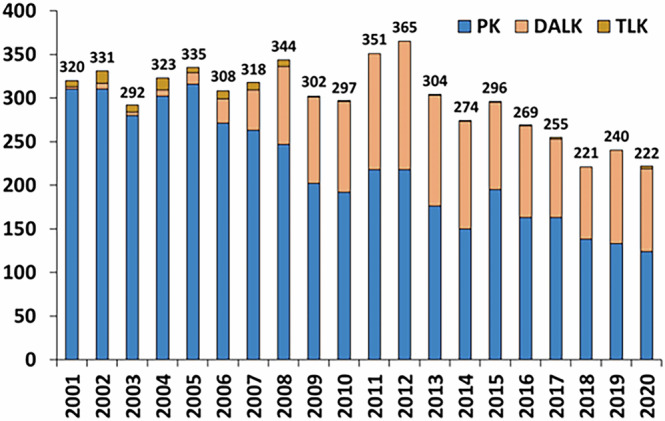
Number of corneal grafts performed for keratoconus in Australia from 2001 to 2020 [17]. DALK deep anterior lamellar keratoplasty, PK penetrating keratoplasty, TLK therapeutic lamellar keratoplasty.

Over the past decade in Australia, data from the Australian Corneal Graft Registry [17] indicated that there has been a decrease in the total number of corneal grafts reported for keratoconus, which included both penetrating keratoplasty (PK) and deep anterior lamellar keratoplasty (DALK). The report found a statistically significant decrease in the combined number of grafts (PK and DALK) from 3170 grafts in 2001–2010 to 2797 grafts in 2011–2020 (*p* < 0.001). DALK has increased in use for keratoconus treatment over time, from around 20% in 2007 to nearly 50% of all corneal grafts for keratoconus in 2020. Figure 1 summarises the number and type of corneal grafts performed for keratoconus in Australia from 2001 to 2020.

### Rigid Gas Permeable Contact Lens Use

**Fig. 2:**
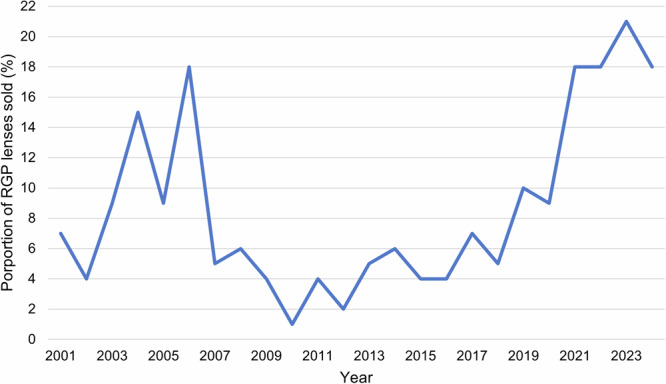
Percentage of RGP contact lenses sold as a proportion of all contact lens sales in Australia from 2001 to 2024 [20]. RGP rigid gas permeable.

**Fig. 3:**
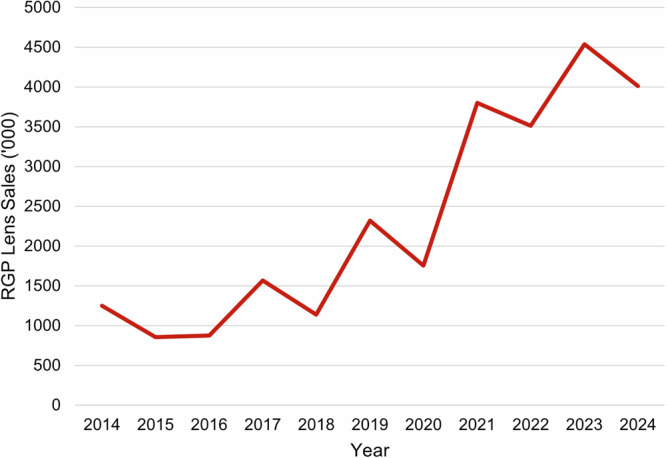
Number of RGP contact lenses sold in Australia from 2014 to 2024 [20]. RGP rigid gas permeable.

**Fig. 4:**
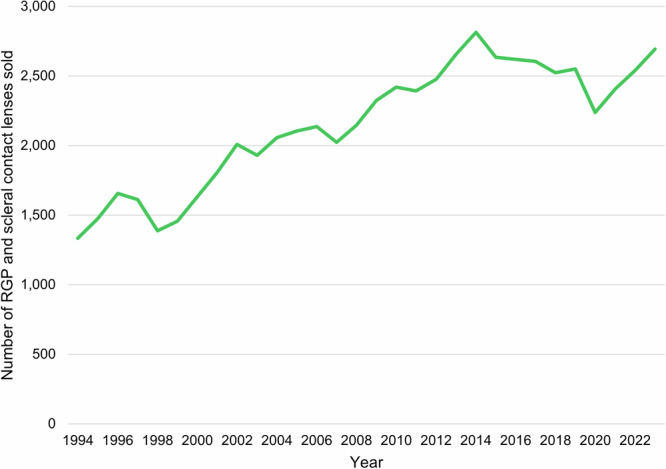
Number of RGP and scleral contact lenses sold for treatment of keratoconus in Australia from 1994 to 2024 [18]. RGP rigid gas permeable.

Figure 2 shows the percentage of RGP contact lenses sold as a proportion of all contact lens sales in Australia from 2001 to 2024. Between 2006 and 2007, the proportion of RGP lenses sold in Australia decreased from 18% to 5%. From 2007 onwards, the overall trend in the proportion of RGP contact lenses sold in Australia has increased from 5% in 2007 to 18% in 2024. Additionally, scleral lenses have grown in popularity, increasing from 8% of all RGP lens fits in 2015 to 41% in 2022. Figure 3 displays the absolute number of RGP contact lenses sold in Australia from 2014 to 2024. The number of RGP lenses sold in Australia per year has increased since 2014, from 1.3 million to 4 million, with a significant increase between 2020 and 2021. Figure 4 illustrates the number of RGP and scleral lenses sold for the management of keratoconus in Australia from 1994 to 2024. Thus, over the past three decades, the number of RGP and scleral lenses sold for treatment of keratoconus has doubled. Using additional data obtained from three major Australian contact lens manufacturers, it is estimated that in 2023 and 2024, approximately 2760 to 4260 RGP and scleral lenses have been sold per year for keratoconus patients.

### Corneal Crosslinking

Use of the item number for CXL procedures in Australia has increased since the addition of CXL to the MBS in 2018. Queensland has the largest number of CXL procedure claims on the MBS, followed by Victoria and New South Wales. As a proportion of their respective populations, Queensland shows the greatest number of CXL procedures on the MBS, followed by South Australia and Victoria. Table 1 summarises the number of CXL procedures performed in Australia each year, categorised by state. Table 2 displays the number of CXL procedures as a proportion of the Australian population.

**Table 1:** Number of corneal crosslinking procedures by year and state in Australia.

Year	NSW	VIC	QLD	SA	WA	TAS	ACT	NT	Total
2018	303	327	349	109	145	28	7	4	1272
2019	490	537	546	178	202	51	17	4	2025
2020	524	656	656	213	233	24	17	1	2324
2021	587	701	644	206	198	33	17	2	2388
2022	563	633	565	151	211	45	32	11	2211
2023	587	538	664	164	187	52	28	4	2234
2024	561	586	701	176	198	68	24	4	2318
Total	3615	3978	4125	1197	1374	301	142	30	14,772

Key: *ACT* Australian Capital Territory, *NSW* New South Wales, *NT* Northern Territory, *QLD* Queensland, *SA* South Australia, *TAS* Tasmania, *VIC* Victoria, *WA* Western Australia.

**Table 2:** Number of corneal crosslinking procedures per 100,000 population from 2018 to 2024 in Australia.

NSW	VIC	QLD	SA	WA	TAS	ACT	NT	Total
43	60	77	66	49	54	31	12	56

Key: *ACT* Australian Capital Territory, *NSW* New South Wales, *NT* Northern Territory, *QLD* Queensland, *SA* South Australia, *TAS* Tasmania, *VIC* Victoria, *WA* Western Australia.

Since the introduction of CXL in Australia, The Save Sight Keratoconus Registry has recorded 2903 CXL procedures in Australia, with 1191 procedures before 2018 and 346, 348, 281, 268, 162, 140 and 167 procedures annually from 2018 through 2024, respectively. The mean age of CXL patients in the Save Sight Keratoconus Registry was 26.2 ± 10.1 years (range 9 to 74 years), with CXL reported to be 2.4 times more common in males than females.

**Fig. 5:**
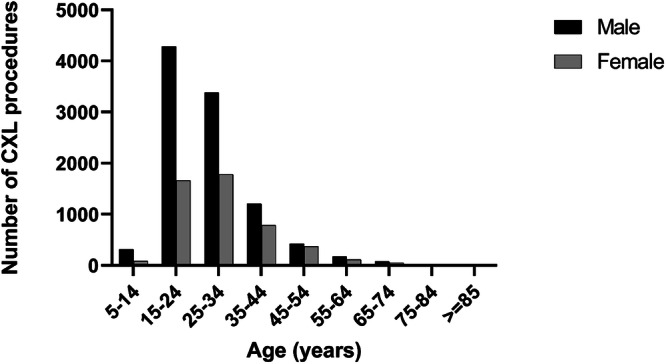
Sex and age distribution of the corneal cross-linking procedures recorded by the Medicare Benefits Scheme in Australia from May 2018 to December 2024 [18]. CXL corneal cross-linking.

**Fig. 6:**
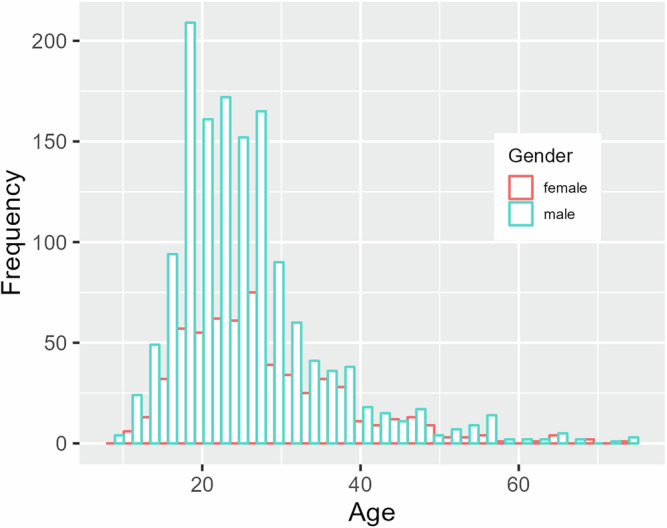
Sex and age distribution of corneal crosslinking procedures in Australia according to the Save Sight Keratoconus Registry data.

Figure 5 shows the number of CXL procedures recorded on the MBS in Australia, stratified by sex and age from May 2018 to December 2024. In terms of age, the number of CXL procedures performed from May 2018 to December 2024 was highest in the 15 to 24 year age group (5949 procedures), followed by the 25 to 34 years (5170 procedures) and 35 to 44 year age group (1997 procedures). The total number of CXL procedures was twice as high in males than females (9894 vs 4878 procedures). In males, the 15 to 24 year age group had the highest number of CXL procedures (4285 procedures), whereas in females, CXL procedures were most prevalent in the 25 to 34 year age group (1785 procedures). Figure 6 illustrates the age at which CXL was performed in Australia for males and females based on the Save Sight Keratoconus Registry data. The histogram also shows that on average, males were undergoing CXL at a higher frequency and younger age compared with their female counterparts.

The prevalence of keratoconus has previously been estimated to be 1.2% in Australian 20-year-olds [5]. Progression may occur in 6% of keratoconus patients in this age group [21]. Given that there are 3.2 million Australians aged 15 to 24 years [22], approximately 2304 CXL procedures should have been performed in this age group. Additionally, since the prevalence of keratoconus has been reported to be twice as high in males than females [23], one would predict twice as many CXL procedures having been performed on males.

### Patient Reported Outcomes in Keratoconus

In Australia, five studies have explored the impact of keratoconus on the QoL using various questionnaires [15, 16, 24–26]. After adjustment for visual acuity, keratoconus patients had approximately 40% lower overall visual function and emotional scores on the Impact of Vision Impairment (IVI) questionnaire [27], when compared to patients with age-related macular degeneration, retinal vein occlusion or diabetic macular oedema [25]. This emphasised that the impact of QoL by keratoconus may be disproportionate to vision loss. The decline in vision-related QoL using the IVI questionnaire was also evidenced by Tan et al., in which reading, mobility and emotional well-being showed significant decline with worsening keratoconus severity [15]. The IVI assesses vision-related impacts on QoL, and is not specific for keratoconus [26]. Two large-scale studies evaluated factors affecting QoL in Australian keratoconus patients using the Keratoconus Outcomes Research Questionnaire (KORQ) [16, 24]. The KORQ is a patient-reported outcomes measure (PROM) validated for keratoconus patients [28]. Kandel et al. found that keratoconus patients who were female, wore contact lenses, had greater disease severity and reduced visual acuity, showed worse activity limitation and emotional scores [16]. Additionally, self-reported visual function and symptoms were more affected by visual acuity in the better eye [16]. These findings were supported by Sahebjada et al [29]. CXL was associated with better activity limitation, symptoms and emotional scores, highlighting its importance in the management of keratoconus [24].

## Discussion

These results highlighted trends in the management and burden of keratoconus over the past few decades in Australia. A steady decrease in the number of corneal transplants for keratoconus was noted, with a greater reduction in PK compared to DALK. This change coincided with an increase in CXL, as well as RGP and scleral contact lens use over the same time period. CXL was more commonly performed in males and in those aged 15 to 24 years, with variability across the states of Australia. Decreased reporting of cross-linking by the Save Sight Keratoconus Registry occurred during the COVID pandemic, with some increase being seen since then, alongside increasing global data [24]. The proportion of scleral lens fitted has increased at a greater rate compared with corneal RGP lenses. Additionally, the total number of RGP and scleral lenses prescribed for keratoconus patients has doubled since 1994.

These changes in the management of keratoconus are consistent with international studies, which have shown a downward trend in corneal transplantation and increased CXL and RGP contact lenses, which are commonly used for the condition [11, 12, 30–32]. Further, the number of corneal transplants in Australia decreased by approximately 30% over a 20 year period [17]. A faster decline in the number of corneal transplants for keratoconus was observed since the introduction of CXL to the MBS in 2018, which eliminated the significant out-of-pocket cost for the procedure. These findings regarding the reduction in keratoplasty for keratoconus since the introduction of CXL are similar to reports from other countries [11, 12, 14]. For example, two studies conducted after the introduction of CXL in Norway found that the number of corneal transplants decreased by 50% after 8 years [12] and 80% after 15 years [14]. In the 7 years after CXL was introduced, the Dutch National Organ Transplant Registry reported that the number of corneal transplants decreased by 25% [11]. Additionally, the 2019 report from the Canadian graft registry found that the number of transplants overall did not decrease 15 years after the introduction of CXL, but the proportion of transplants performed for keratoconus dropped significantly [33]. Other surgical techniques, such as intracorneal ring segments (ICRS) [34] and corneal allogeneic intrastromal rings (CAIRS) [35], have also gained traction as alternatives for keratoconus treatment that may impact corneal graft trends in the future. ICRS flatten the central cornea by the arc-shortening effect on the corneal stroma, and offers a viable means to improve vision in keratoconus patients, especially for those unsuitable for CXL or awaiting corneal grafting. Previously, manual tunnel dissection for ICRS was associated with multiple adverse events [34]. Nowadays, femtosecond laser-assisted tunnel creation offers proven safety and efficacy with significantly reduced complication rates [34]. Further, combining CXL with ICRS results in additive visual and refractive benefits [36, 37]. CAIRS is a relatively novel procedure which involves implanting ring segments from donor corneal stromal tissue into stromal channels [35]. They were developed as a biological alternative to synthetic ICRS to improve corneal stability and reduce astigmatism while minimising the risks involved with implanting synthetic material [35]. A recent systematic review found that CAIRS transplantation significantly improved visual acuity and reduced keratometry values, with a low risk of post-operative complications [38]. Although promising, further trials are needed to compare the effectiveness of CAIRS with other therapies for keratoconus. There are currently no Australian data on utilisation trends for ICRS and CAIRS. As uptake grows, prospective studies should evaluate their real-world outcomes. The increase in CXL procedures can be attributed to its effectiveness in stabilising keratoconus compared to other forms of treatment. Studies assessing the outcomes of CXL in keratoconus have demonstrated short and long-term reductions in keratometry (*K*_max_) and improvement in visual symptoms and visual acuity [39–42]. CXL also leads to improvements in the QoL in keratoconus patients [24]. The Save Sight Keratoconus Registry found that patients with keratoconus who underwent CXL had improved mean activity limitation, symptoms and emotional scores as measured using the KORQ after 6 months [24]. This is supported by Pinto et al. and Ferrini et al., who also found improved QoL scores using the KORQ [43, 44]. In patients with keratoconus, Labiris et al. noted that the mental health and dependency scores improved after CXL [45], while Cingu et al. demonstrated that CXL was associated with improved vision-related QoL and reduced anxiety [46]. These improvements in QoL and psychological well-being appear to be long-lasting, as they have been shown to persist for 4 years after the initial CXL procedure [47].

The current data on CXL indicate that the procedure is predominantly performed in keratoconus patients between 15 and 34 years of age, and males are twice as likely to undergo CXL as females. This is consistent with the literature, which states that CXL is typically performed in this age range due to the propensity for disease progression [48–50]. This age of treatment is also consistent with data from several studies using the Save Sight Keratoconus Registry, a large web-based database enroling patients from five different countries [24, 39, 51]. This reflects current clinical practice in which CXL is indicated in patients with documented progression of keratoconus, which is more common in younger patients [52]. Additionally, males have an earlier onset, faster progression and higher prevalence of keratoconus than females, which may explain the higher frequency and earlier age of CXL for males reported here [4, 21, 53]. It has been suggested that sex hormones may influence the cornea in a way that results in earlier onset and more rapid progression of keratoconus in males, and could also account for the higher prevalence [54]. Based on the current estimates of young adults living in Australia, CXL was being performed at the expected rate across the sexes, with twice as many procedures being performed on males than females. The present study also suggests that Australia has a good coverage rate for CXL, as the total number of CXL procedures was greater than the calculated expected rate. However, there was significant state-to-state variability in the number of CXL procedures, particularly in the Northern Territory, Australian Capital Territory and New South Wales, where the rates of CXL were lower than in other states. The lower rates of CXL in these states may reflect a combination of reduced service capacity, longer travel distances and differences in access to public CXL services. In the Northern Territory and rural New South Wales, there is limited access to corneal specialists, and individuals often have to travel long distances at their own expense and find time for an appointment [55]. It may be beneficial to dedicate further resources to these areas to improve access and uptake of CXL for keratoconus patients. A need to monitor younger patients with keratoconus may also be required, as the risk of progression is greater in younger individuals [56, 57].

Targeted education can assist with earlier diagnosis and treatment uptake in keratoconus. Awareness of early symptoms and risk factors, including habitual eye rubbing and atopy, remains low in many settings, thereby delaying presentation [58]. There is substantial evidence associating eye rubbing with keratoconus, and longitudinal data suggest that counselling to cease rubbing can stabilise the disease in a substantial proportion of eyes [59, 60]. Educational messages should emphasise prompt assessment for progressive blur, avoidance of eye rubbing and proactive allergy control, alongside clear referral pathways to clinics with tomography and cross-linking capability.

The advancement in contact lens technology is also likely to have contributed to the decreasing use of keratoplasty for keratoconus. Many keratoconus patients who previously underwent corneal transplantation are now experiencing good visual outcomes with rigid contact lenses, including scleral lenses. This is evidenced by a large study highlighting improved quality of life by keratoconus patients treated using contact lenses [61]. Between 1994 and 2024, the current study noted an increase in the number of RGP and scleral lenses prescribed for keratoconus patients, from 1334 to 2692 lenses. A dramatic increase in the proportion of scleral lenses sold over the past decade was also found, i.e., from 8% in 2015 to 41% in 2022. This is similar to a recent study in Chicago which found that the percentage of keratoconus patients using scleral lenses increased from 0% in 2010 to 22% in 2020 [62]. The greater uptake of scleral lenses is likely due to the fact that new scleral lens designs exhibit greater comfort levels and stability compared with corneal RGP lenses for patients with keratoconus [63]. While previously reserved for keratoconus patients with the most severe disease, the availability of improved materials and designs that are easier to fit has enabled scleral lenses to become more accessible to healthcare professionals [64]. Although corneal RGP lenses remain the primary lens of choice for keratoconus, scleral lenses are being increasingly recognised as a suitable alternative, particularly for more severe disease. Furthermore, recent advancements in hybrid and custom-designed contact lenses for keratoconus also have the potential to alter the therapeutic threshold for surgical intervention. Hybrid lenses consist of a rigid lens material that corrects corneal irregularity and a soft lens material that provides lens centration and peripheral comfort. Although studies on hybrid lenses are limited, they have been shown to have similar visual quality but increased vision-related QoL scores compared to RGP lenses in keratoconic patients [65, 66]. Custom-designed soft contact lenses have also gained momentum in recent years due to the increase in technological capacity and increased knowledge on optical aberrations in keratoconus. Limited studies on these lenses have shown good visual results in keratoconus patients who are intolerant to RGPs [67, 68].

The current data showed that the overall proportion of RGP lenses sold in Australia is rising, from 5% in 2007 to 18% in 2024. The increased use of RGP lenses was also captured in a 2015 survey of 71 optometrists in Australia, with 9.2% of participants reporting prescribing RGP contact lenses daily, while 47.7% prescribed them at least once a month [69]. Although the use of contact lenses for keratoconus is becoming more widespread, the aforementioned study highlighted some barriers that optometrists experience when prescribing RGP lenses, including a lack of experience with fitting these lenses, the time taken for fitting and limited access to corneal topography [69]. These reasons were echoed by optometrists in Latin America, USA, UK and Spain [70]. Thus, further training for optometrists with regard to fitting RGP lenses in patients with keratoconus, and the provision of more corneal topography devices, would help facilitate the use of contact lenses for the management of keratoconus. Since the cost of contact lenses for patients with keratoconus is not covered by the government in Australia, measures to increase their affordability would also likely increase rates of wear.

Keratoconus has a significant impact on QoL, and the use of patient-reported outcome measures (PROMs) enables the benefits of treatments to be understood. Widely used non-keratoconus-specific instruments such as the National Eye Institute Vision Function Questionnaire (NEI-VFQ) [71] and IVI [27] lack content specific to keratoconus [16]. Consequently, disease-specific measures like the KORQ, which was developed and validated using Rasch analysis, are better suited to capture the full impact of keratoconus on visual function and guide symptom management [16]. Although the KORQ does not measure psychosocial well-being and inconveniences, it is currently the recommended questionnaire to measure keratoconus outcomes [72]. Studies involving the SSKR have provided important insights into the impact of keratoconus on QoL in keratoconus patients using the KORQ [16, 24, 28, 39]. However, there is a limited collection of PROMs in clinical practice and a need for broader implementation of keratoconus-specific tools. Further work should also be performed to improve existing PROMs, as they currently do not provide a comprehensive overview of all QoL domains [28, 43].

The main strength of this study was the large sample size obtained using information from a variety of national databases. It is a unique study providing an overview of the changes in management and burden of keratoconus in Australia over a long time period. A limitation was that there was no comprehensive single data source that reported on trends in keratoconus management. For example, although including the majority of corneal grafts and CXL, the ACGR and MBS data, respectively, may not encompass all procedures performed in Australia. Underreporting may occur as some corneal surgeons may not contribute to the ACGR, and the limited CXL performed in public hospitals may not utilise MBS subsidies. Another limitation was selection bias in voluntary registries or surveys such as the SSKR and International Contact Lens survey [20], as not all Australian eyecare professionals may have participated in the SSKR, and those that responded may have had a particular interest in CXL or contact lenses. Clinicians, researchers, patients and health policy officials would benefit from comprehensive data on the trends in the management and burden of keratoconus. Critically, treatment outcomes, including PROMs, should be analysed and reported to drive improvements in care.

In Australia, available data indicate a decreasing number of keratoplasties being performed for keratoconus, along with increases in CXL and contact lens use. Future studies should evaluate the outcomes associated with the change in keratoconus management patterns. Longitudinal outcome studies comparing early versus delayed intervention strategies (especially with CXL), access to emerging treatments such as CAIRS or topography-guided procedures and integration of AI-based decision support would also be useful in guiding management techniques. Registries provide a means for collecting up-to-date clinical and patient-reported outcome data.

## Data Availability

No datasets were generated or analysed during the current study.
